# Identification of putative drought-responsive genes in rice using gene co-expression analysis

**DOI:** 10.6026/97320630015480

**Published:** 2019-07-31

**Authors:** Yanmei Lv, Lei Xu, Komivi Dossa, Kun Zhou, Mingdong Zhu, Hongjun Xie, Shanjun Tang, Yaying Yu, Xiayu Guo, Bin Zhou

**Affiliations:** 1 Hunan Rice Research Institute, Changsha, 410125, China; 2 College of Pharmacy, Hubei University of Chinese Medicine, China; 3 Wuhan Benagen Tech Solutions Company Limited, Wuhan 430070, China; 4 State key laboratory of hybrid rice, Changsha, 410125, China; 5 Laboratory of Indica Rice Genetics and Breeding in the Middle and Lower Reaches of Yangtze River Valley, Ministry of Agriculture,Changsha, 410125, China

**Keywords:** drought, transcriptome, WGCNA, co-expressed genes, network analysis

## Abstract

Drought is one of the major abiotic stresses causing yield losses and restricted growing area for several major crops. Rice being a major staple food crop and sensitive to water-deficit conditions bears heavy yield losses due to drought stress. To breed drought tolerant rice cultivars, it is of interest to understand the mechanisms of drought tolerance. In this regard, large amount of publicly available transcriptome datasets could be utilized. In this study, we used different transcriptome datasets obtained under drought stress in comparison to normal conditions (control) to identify novel drought responsive genes and their underlying molecular mechanisms. We found 517 core drought responsive differentially expressed genes (DEGs) and different modules using gene co-expression analysis to specifically predict their biological roles in drought tolerance. Gene ontology and KEGG analyses showed key biological processes and metabolic pathways involved in drought tolerance. Further, network analysis pinpointed important hub DEGs and major transcription factors regulating the expression of drought responsive genes in each module. These identified novel DEGs and transcription factors could be functionally characterized using systems biology approaches, which can significantly enhance our knowledge about the molecular mechanisms of drought tolerance in rice.

## Background

Rice is a model plant species and a major staple food crop among cereal feeding around half of the world population [1]. Since, rice has very high-water requirements for good production, drought is a major cause for its yield reduction and limits the growing area worldwide [2]. Several projects were launched for the annotation of rice genome which enabled researchers to utilize the genomeannotation information for the functional characterization of rice genes. One of the promising projects is Rice Genome Annotation Project by MSU (http://rice.plantbiology.msu.edu/index.shtml) with its 7th release providing annotation of all the 12 chromosomes and about 40,000-60,000 estimated rice genes [3]. Recently the reduced cost of transcriptome analysis and availability of efficient omics tools have shifted the attention of molecular biologists to utilize the genome annotation information via computational approaches. Since most of the biological pathways involve coordinated regulation of dozens-to-hundreds of genes (known as co-expressed genes), identification of these co-expressed genes provides useful information for the characterization of key genes via systems level approaches. The development of various omics databases such as the gene ontology (GO) consortium, 2017 (http://geneontology.org/) [4], AGRIGO V2.0 (http://systemsbiology.cau.edu.cn/agriGOv2/) [5], and kyoto encyclopedia of genes and genomes (KEGG) (https://www.genome.jp/kegg/), etc. have provided the opportunity to explore the molecular mechanisms and biological pathways of genes involved in biotic and abiotic stresses by utilizing the available large transcriptome datasets with the minimum cost.

In this study, we used several computational and omics tools to understand the molecular components and biological processes involved in drought tolerance in rice. Recently, co-expression network analysis has emerged as a very useful approach for the functional annotation of novel genes [6,7]. It is based on the idea that all the genes involved in a particular biological pathway will be connected to each other in a network. The nodes in the network represent genes and edges represent their connection to other genes. One of the promising applications of network analysis is to identify the functional modules. Weighted gene co-expression network analysis (WGCNA), a package in R, has been used for the network analysis to detect the networks of co-expressed genes and divide the core genome into different modules [8]. These modules can then be used to pinpoint drought responsive key hub genes and predict their putative role in particular biological processes and metabolic pathways, thus facilitate for the elucidation of molecular and biochemical mechanisms using systems biology approaches. For example, using WGCNA analysis of the RNA-seq data, researchers have identified key regulators of flower and fruit development in strawberry [9]. In addition, WGCNA analysis is an important source for the functional annotation of novel genes in rice, thereby providing a helping hand for the functional characterization of novel genes in rice [6]. Loss-of-function mutants have been widely used to characterize various genes for their involvement in particular phenotype and understand the involved molecular mechanisms [10,11]. However, it mostly involves development of large mapping populations for the map-based cloning approach which is a very time taking, laborious and tricky approach. On the other hand, identification of putative target genes involved in particular phenotype or biological process using transcriptome datasets has becoming popular approach for the characterization of genes involved in target phenotype [12]. This can help reduce the long time and large land area needed for the development of mapping populations or near isogenic lines of the specific genes. Here, we identified several putative drought responsive uncharacterized genes using co-expression network analysis from large transcriptomic datasets that will serve as an important resource for the identification of genes involved in drought tolerance using loss-of-function knockout mutants. In addition, we have highlighted several biological processes and metabolic pathways of the identified modules that are involved in drought tolerance by the particular module's genes.

## Methodology

### Plant material and growth conditions

The seeds of japonica rice cultivar 'Hunan' were collected from Hunan Rice Research Institute, Changsha, China. The experiment was conducted in controlled environment in a greenhouse. The seeds were surface sterilized with 1% NaOCl solution to remove the contaminants. Sterilized seeds were immersed in water at 37°C for two days followed by germination at 30°C with a photoperiod of 16 h (light)/8h (dark) and a relative humidity set at 70%. Seedlings were grown for a week in 2000 ml pots containing a½ strength Hoagland nutrient solution. Then, the drought stress treatment was applied by adding to the nutrient solution 20% PEG6000 solution and then the root samples were harvested after 0, 1, 3 and 5 d time period. The control seedlings were maintained in a nutrient solution without drought treatment and samples were collected in parallel. Three biological replicates were maintained for control and drought treatment for each time point.

### RNA isolation and qRT-PCR gene expression analysis

Plant total RNA from control and drought-treated root samples was isolated using an RNA extraction kit (Tiangen), and the first-strand cDNA was synthesized from 2 ng of RNA by reverse transcriptase (Invitrogen, Carlsbad, CA, USA), and then diluted (1:100) for use in qRT-PCR with SYBR Premix ExTaq Mix (Takara, Dalian, Liaoning, China) in a total volume of 15 mL. Reactions were performed in a LightCycler 480 thermal cycler (Roche, Basel, Switzerland), following the manufacturer's instructions. Three biological replicates were performed for each sample, and the expression level was normalized to that of rice Actin-1 gene (LOC4333919), which was used as the endogenous control gene. The primer sequences used in this study are given in supplemental material 1.

### Data acquisition and processing

RNA sequencing data of gene expression profiles under control and drought stress condition of a japonica rice cultivar Nipponbare was downloaded from National Center for Biotechnology Information (https://www.ncbi.nlm.nih.gov/sra). A total of five different datasets (DRP000997, root transcriptome under control and drought stress treatment after 6, 12 and 24 h; SRP052306, leaf transcriptome under control and drought stress treatment; SRP052309, transcriptome analysis of loss of function mutant of OsbHLH148 gene under control and drought stress; SRP052310, transcriptome analysis of loss of function mutant of OsHSFA2e gene under control and drought stress, and SRP075204, transcriptome analysis of loss of function and over-expression mutant of OsHSFA2e gene under control and drought stress) were obtained which consists of 26 samples. The details of accession numbers and samples of all datasets are given in supplemental material 2. The raw data were first processed with FastQC (http://www.bioinformatics.babraham.ac.uk/ projects/fastqc/) to filter out adapters and low-quality sequences. Then, the clean reads were mapped to the rice genome using HISAT [13]. RSEM package was used to calculate gene expression level for each sample expressed as fragments per kilobase of transcript per million fragments mapped (FPKM) [14]. The gene expression levels in stressed samples were compared with those in control samples in order to identify the differentially expressed genes (DEG). The DEGs were detected as previously described [15] based on the parameters: Fold change > = 2.00 and Probability > = 0.8 with a significant false discovery rate-adjusted P value (FDR) <0.05 based on the three biological replicates. Gene Ontology (GO) and Kyoto Encyclopedia of Genes and Genomes (KEGG) enrichment analyses for the DEGs were performed using the clusterProfiler version 3.8.

### 

Weighted gene co-expression network analysis (WGCNA) Gene co-expression networks were constructed using WGCNA package in R software [8]. The core DEGs were further divided into three modules using WGCNA and correlation of each module with drought stress was calculated. Genes in each module that is blue, grey and turquoise are listed in supplemental material 3. Moduletrait associations were estimated using the correlation between the module eigengene and the stress treatments. Network visualization for each module was performed using the Cytoscape software version 3.6.1 with a cut off of the weight parameter obtained from the WGCNA, set at 0.3. The gene co-expression network is a scalefree weighted gene network with multiple nodes connected to different nodes via edges. Each node represents a gene which is connected to different number of genes. The gene which is connected to a greater number of genes is denoted with bigger size and is more important for its interaction with large number of genes. In addition, key transcription factors involved in drought tolerance and their putative target genes were highlighted.

## Results

### Identification of the drought-responsive core DEGs in rice

**Figure 1:**
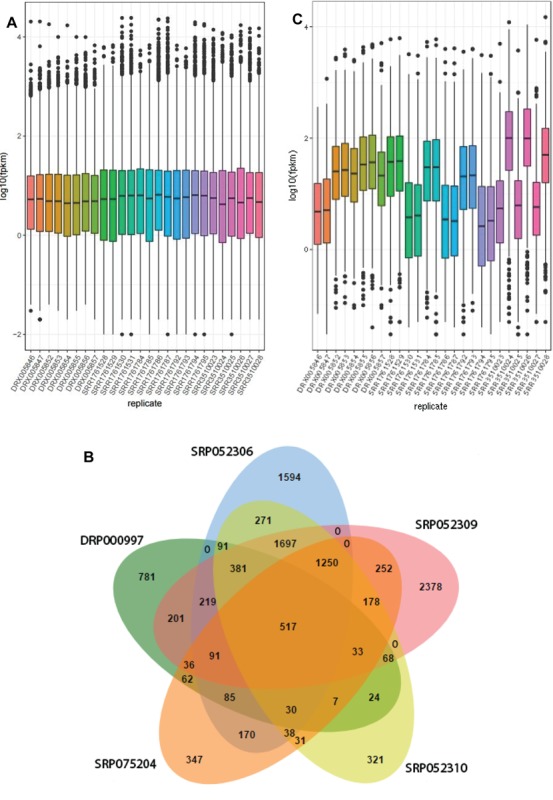
Identification of drought responsive core DEGs in rice. (A) Expression profiles of whole genome datasets based on FPKM values. (B) Venn Diagram showing conserved drought responsive DEGs. (C) Expression profiles of core drought responsive DEGs based on FPKM values.

In this study, we analyzed global gene expression profiles of japonica rice cultivar Nipponbare for drought stress response using different datasets. The datasets contain DRP000997 (root transcriptome under control and drought stress treatment after 6, 12 and 24 h), SRP052306 (leaf transcriptome under control and drought stress treatment), SRP052309 (transcriptome analysis of loss of function mutant of OsbHLH148 gene under control and drought stress), SRP052310 (transcriptome analysis of loss of function mutant of OsHSFA2e gene under control and drought stress) and SRP075204 (transcriptome analysis of loss of function and over-expression mutant of OsHSFA2e gene under control and drought stress). The overall global gene expression profiles are similar among different datasets under drought stress suggesting the compatibility of drought stress response in rice (Figure 1A). A total of 42,189 differentially expressed genes (DEGs) were identified among different samples in the datasets.

**Figure 2:**
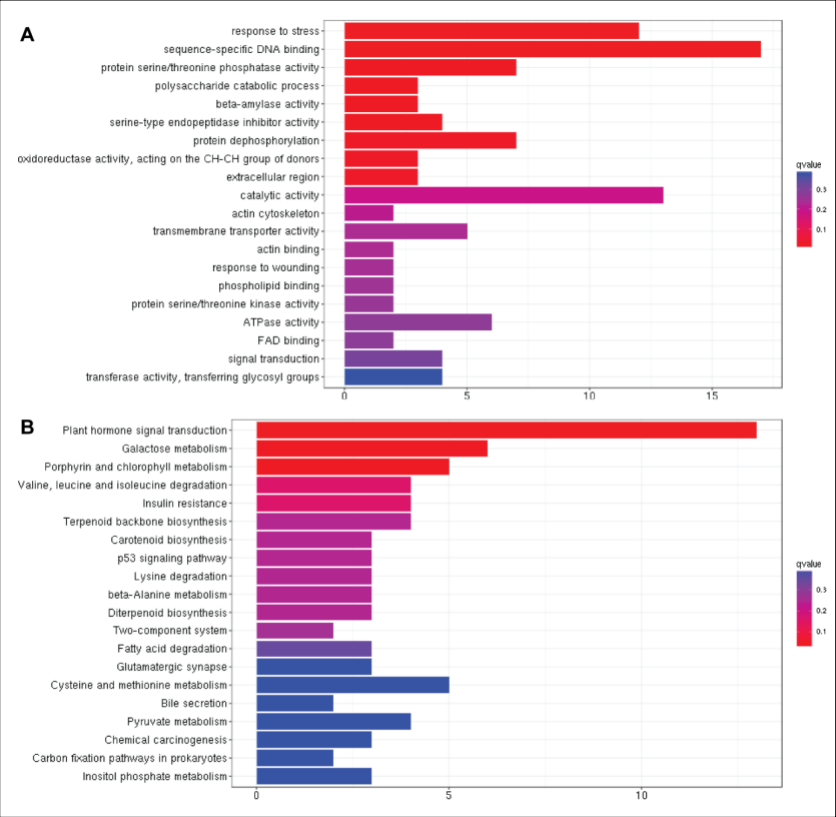
GO (A) and KEGG analysis (B) of core drought responsive DEGs.

In order to identify the drought responsive core DEGs, we crosscompared the DEGs among different datasets used in this study, which displayed 517 core DEGs that are conserved among different drought treatments (Figure 1B). The global gene expression profiles of these core DEGs vary across the datasets and among control and stressed treatments, confirming that these core DEGs are responsive to drought (Figure 1C). We then employed these 517 core DEGs to get further insight into the mechanisms of drought responses. To achieve, we first performed gene ontology (GO) to identify the significantly enriched biological processes contributed by these DEGs. GO analysis unveiled that biological process related to "sequence-specific DNA binding" was the most enriched biological process followed by "response to stress" with the q-value lower than 0.1, suggesting that specific transcription factors may be involved in drought tolerance by regulating the expression of stress-responsive genes (Figure 2A). We then performed kyoto encyclopedia of genes and genomes (KEGG) analysis to identify enriched pathways contributed by 517 core drought responsive DEGs. Plant hormone signal transduction was the most significantly enriched pathway followed by carbohydrate metabolism with 24% and 21% of the core DEGs involved in these pathways, respectively, indicating that particular hormones played key roles in drought tolerance (Figure 2B, supplemental material 4).

### WGCNA of the drought-responsive core genome

**Figure 3:**
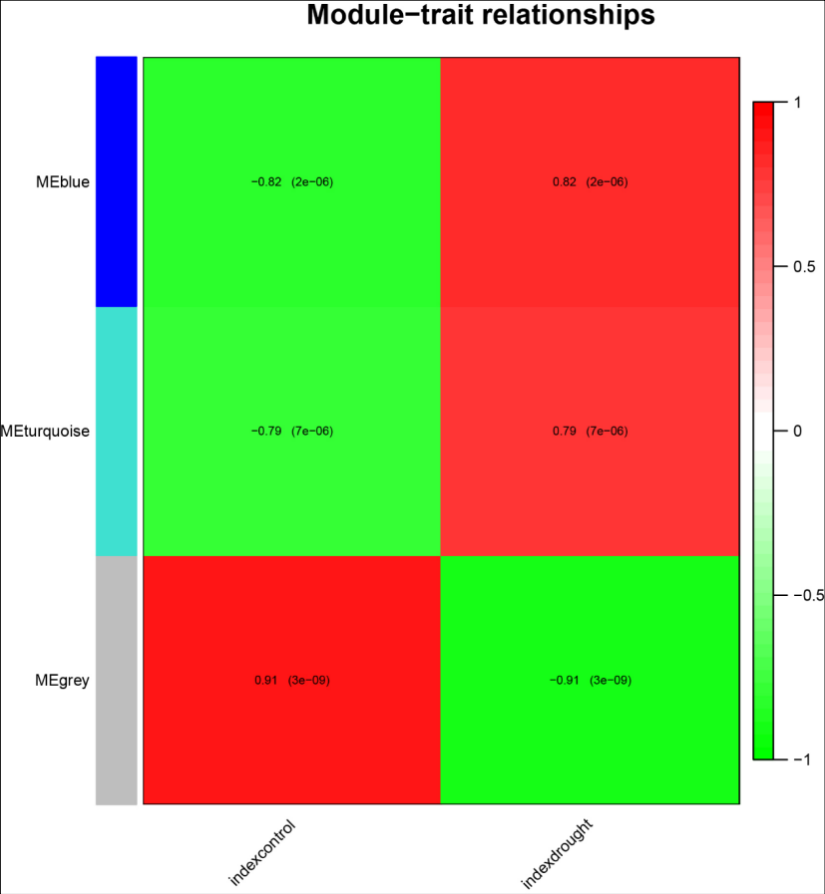
Matrix showing Module-Trait Relationships (MTRs) of different modules under control and drought stress.

In order to identify the different modules involved in drought tolerance in rice, we performed WGCNA which divided the core 517 hub DEGs into three modules; blue, grey and turquoise (Figure 3). Blue module contains 218 DEGs, grey module contains 76 DEGs and turquoise module contains 223 DEGs (supplemental material 3). Blue and turquoise modules had the positive correlation (r=0.82 and r=0.79, respectively) with drought stress suggesting that genes in these modules positively regulate the drought tolerance in rice (Figure 3). However, grey module had the negative correlation (r=- 0.91) with drought stress which indicates that genes in this module negatively regulate the drought tolerance in rice, and thus should be down-regulated under drought stress in order for the plant to survive the drought stress (Figure 3). Hence, it can be conferred that biological pathways enriched in grey module will be different from blue and turquoise modules.

**Figure 4:**
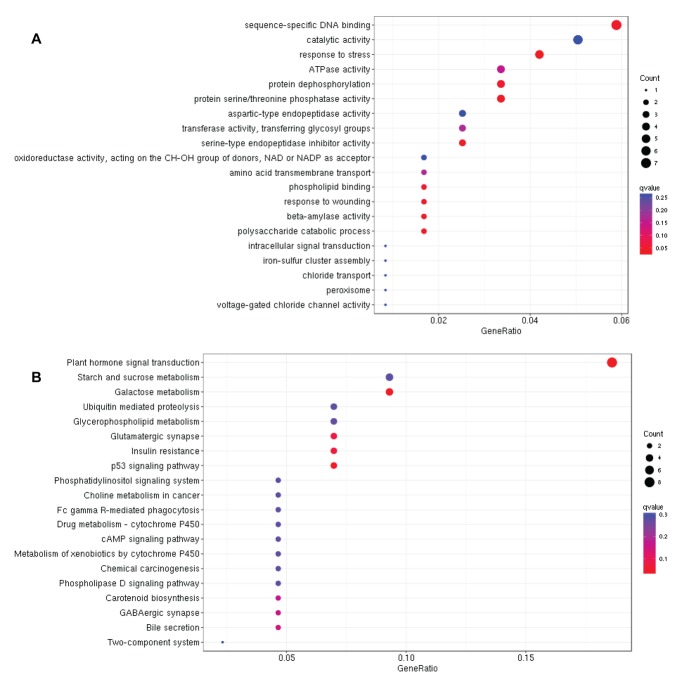
Gene ontology (A) and KEGG analysis (B) of DEGs belonging to blue module under drought stress in rice.

**Figure 5:**
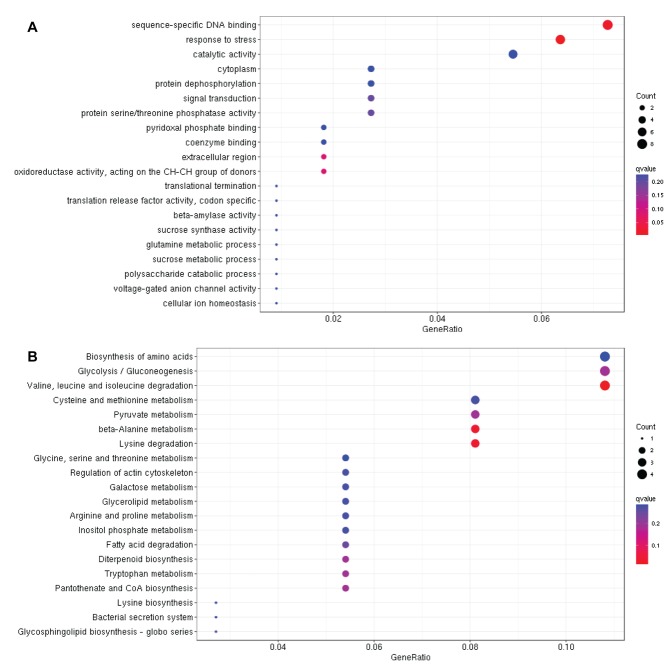
Gene ontology (A) and KEGG analysis (B) of DEGs belonging to turquoise module under drought stress in rice.

We further expanded the study by performing the GO and KEGG analyses of each module to identify its involvement in particular biological process and metabolic pathway. GO analysis of the 218 DEGs belonging to blue module displayed "sequence-specific DNA binding" as the most enriched biological process, suggesting that transcription factors may be involved in drought tolerance by regulating the expression of drought responsive genes (Figure 4A). Moreover, KEGG analysis of these DEGs identified "plant hormone signal transduction" as the most enriched metabolic pathway followed by carbohydrate metabolism related pathways (Figure 4B). Similarly, GO and KEGG analysis of the 223 turquoise DEGs identified "sequence-specific DNA binding" as the most enriched biological process and "plant hormone signal transduction" as the most enriched metabolic pathway, similar to the blue module genes suggesting that blue and turquoise modules may participate in the same biological processes and metabolic pathways (Figure 5).

**Figure 6:**
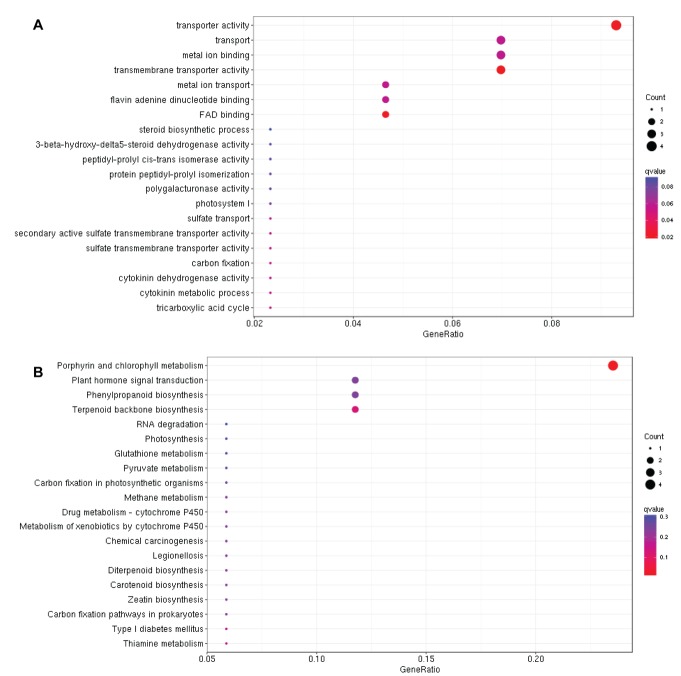
Gene ontology (A) and KEGG analysis (B) of DEGs belonging to grey module under drought stress in rice.

In contrast, GO analysis of the 76 DEGs belonged to grey module detected "transporter activity" as the most enriched biological process under drought stress (Figure 6A). Further KEGG analysis revealed that "porphyrin and chlorophyll metabolism" was the most significantly enriched metabolic pathway suggesting that photosynthesis rate might be affected under drought stress probably due to closing of stomata to avoid water loss (Figure 6B).

### Networks displaying relationships among genes within coexpressed modules

**Figure 7:**
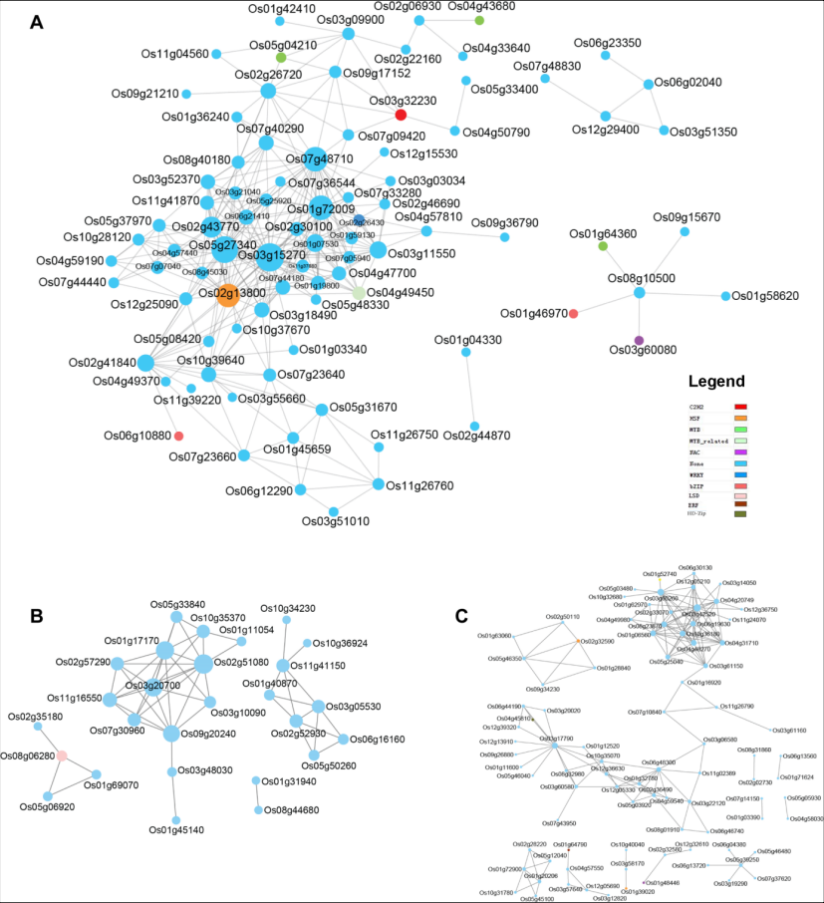
Co-expression network analysis of blue (A), turquoise (B) and grey (C) modules. The size of node circle is positively correlated with the number of genes it interacts.

We further extended the study to network analysis of detected modules to identify key hub genes and their correlation. Genes in the blue module were divided into four clusters, each having network of different number of genes (Figure 7A). Genes encoding transcription factors (TFs) are represented with different node colors except sky blue. The size of node circle is positively correlated with the number of genes it interacts.

The key genes in the blue module network include LOC_Os03g15270 (carboxyesterase 17), LOC_Os05g27340 (DUF567 domain containing protein), LOC_Os01g72009 (no functional annotation) and LOC_Os07g48710 (calmodulin (CAM)-binding protein). In addition, it also includes several genes encoding TFs such as heat-shock transcription factor (LOC_Os02g13800), WRKY DNA-binding protein 11 (LOC_Os02g26430), Homeodomain-like superfamily protein (LOC_Os04g49450), MYB (LOC_Os05g04210 and LOC_Os04g43680), bZIP (LOC_Os06g10880) and C2H2 transcription factor (LOC_Os03g32230) (Figure 7A). Key genes in the grey module included LOC_Os03g20700 (Mg-protoporphyrin IX chelatase, CHLH), LOC_Os02g51080 (Pyridine nucleotide-disulfide oxidoreductase family protein) and LOC_Os01g17170 (dicarboxylate diiron protein) (Figure 7B). In addition, it also includes a zinc finger domain TF (LOC_Os08g06280) which interacts with three genes namely LOC_Os01g69070 (Auxin efflux carrier family protein), LOC_Os02g35180 (OsRR2 type-A response regulator) and LOC_Os05g06920 (Ca2+-activated RelA/spot homolog) (Figure 7B).

Key genes in the turquoise module were LOC_Os03g42520 (uncharacterized protein), LOC_Os03g60260 (aromatic and neutral transporter 1), LOC_Os06g19630 (Steroid nuclear receptor, ligandbinding), LOC_Os03g17790 (Low temperature and salt responsive protein family), LOC_Os10g36180 (CAP160 protein) and LOC_Os04g31710 (uncharacterized protein) (Figure 7C). Further, it also had three TFs namely DUF584 domain containing protein (LOC_Os01g52740), homeobox 7 (LOC_Os04g45810) and heat shock transcription factor A3 (LOC_Os02g32590). Since several transcription factors particularly heat shock transcription factors (HSF), WRKY, myeloblastosis (MYB) and basic leucine zipper domain (bZIP) have been well reported to regulate the expression of key genes involved in drought tolerance, the novel TFs identified and presented in this study could be potential players of drought tolerance in rice.

### qRT-PCR validation of selected genes from each module under drought stress

We selected 16 genes from the three modules and performed a qRTPCR analysis of their gene expression after 5 d under drought stress simulated by PEG6000 treatment in an independent rice cultivar 'Hunan'. The results showed that the expression levels of all the selected genes were significantly changed (|FC>2|) at different time points under drought stress (supplemental material 5). This confirms that the genes identified through our computational analysis are well responsive to drought in rice.

## Discussion

Drought is a major abiotic stress that negatively affects crop production in major crops including rice [16], wheat [17], maize [18, 19] and cotton [20]. Rice being a major staple food crop is particularly sensitive to drought stress due to its large water requirements. Thus, understanding the molecular mechanisms of drought tolerance in rice is particularly important to cope with the challenge. Transcriptome-based studies have provided a new platform to understand the molecular mechanisms and biological processes involved in drought tolerance. These RNA-seq data generates a bundle of information for a target phenotype or stress, however, resourceful utilization of this data has been a bottleneck. Recently, availability of several bioinformatics and statistical tools have helped the researchers to pinpoint key biological processes and metabolic pathways involved in biotic or abiotic stress tolerance, thus efficiently utilizing the RNA-seq data [12,21].

In this study, we aimed at understanding the key players of drought tolerance in rice using efficient statistical and bioinformatics tools by utilizing large transcriptome datasets. Datasets contain gene expression profiles from RNA-seq data of japonica rice cv. Nipponbare under control and drought stress conditions. WGCNA is a powerful package in R which divides the core DEGs in different modules based on correlation among coexpressed genes involved in particular metabolic pathway [8]. In the present investigation, we first identified drought responsive core DEGs by cross comparison of various transcriptome datasets of rice under drought stress (Figure 1). This core droughtresponsive DEGs were subjected to gene ontology enrichment which unveiled "sequence-specific DNA binding" as the most enriched biological process in drought tolerance in rice (Figure 2A). Transcription factors have been well documented to pl ay key role in stress tolerance by regulation of stress-responsive gene expression [22]. The TFs work by binding to the promoter region of particular genes and thus regulate their expression. Thus, GO analysis highlighted the possible role of TFs in drought tolerance in rice. KEGG analysis further revealed "plant hormone signal transduction" as the most enriched metabolic pathway of these core DEGs which further extended our understanding of the metabolic pathways involved in drought tolerance in rice (Figure 2B). Later, we did WGCNA of these core DEGs that divided them into three modules, each of them contributes to drought tolerance in a unique metabolic pathway. Modules blue and turquoise had positive correlation with drought stress treatment, thus genes in these modules positively regulate drought tolerance in rice. On the other hand, grey module had a negative correlation with drought stress, thus genes in this module negatively regulate drought tolerance in rice (Figure 3).

To get a deeper insight of how these modules participate in drought tolerance, we performed GO and KEGG analysis of each module separately. Results revealed that "sequence-specific DNA binding" is the most enriched biological process in blue and turquoise modules, suggesting that TFs are involved in drought tolerance. In addition, both of these modules play role in drought tolerance in similar pathway and genes in these pathways positively regulate drought tolerance. However, GO analysis revealed transporter activity as the most enriched biological process in grey module and genes in this module negatively regulate drought tolerance (Figure 5). In addition, KEGG analysis showed that "porphyrin and chlorophyll metabolism" was the most enriched metabolic pathway in grey module. Porphyrin is an important metabolite and its production under drought stress is critical for stress tolerance in rice. Rice plants over-expressing proto porphyrinogen oxidase showed improved drought tolerance under limited water supply [23]. Similarly, heat and drought stresses also affect the rate of photosynthesis in plants due to closing of stomata and reduced chlorophyll contents [18,24,25]. Genes involved in sustained photosynthesis under drought stress would be imperative to play role in drought tolerance in rice. Since grey module genes negatively regulate drought tolerance, it could be suggested that these genes would be playing role for the negative regulation of "porphyrin and chlorophyll metabolism" under drought stress in rice. However, down regulation of these genes could be utilized for the improvement of drought tolerance in rice.

Since TFs have been well characterized to be involved in abiotic stress tolerance, we further extended the study to identify key hub genes and TFs in each module. Network analysis is a very useful tool to pinpoint association among different genes particularly those playing role in a certain pathway [26]. It also delineates the putative interaction among TFs and their target genes. HSFs are a big class of TFs having important role in abiotic stress tolerance particularly drought and heat stress via regulating the expression of drought and heat stress responsive genes [27,28]. Identification of putative HSF genes responsive to drought stress in rice would improve our knowledge about the mechanism of drought tolerance in rice. In this study, network analysis identified two novel uncharacterized HSF genes (LOC_Os02g13800 and LOC_Os02g325900), which showed interaction with several genes in the blue and turquoise module, respectively. These genes could be the putative target genes involved in drought stress response (Figure 7). WRKY TFs are well known TFs involved in biotic and abiotic stress tolerance in plants [29]. In this report, we identified a novel WRKY TF (LOC_Os02g26430) interacting with several genes of blue module and had highest expression in reproductive tissues including panicle and anthers in rice (http://ricexpro.dna.affrc.go.jp/). Since rice is most susceptible to drought and heat stress at reproductive stage, thus this could be a potential TF regulating drought tolerance in rice at reproductive stage [1,30]. In addition, Homeodomain, MYB, bZIP and C2H2 TFs were shown to be involved in drought tolerance in crop plants by modulating the expression of several genes [31-33]. Here, we report novel drought responsive TFs belonging to blue module, including Homeodomain protein (LOC_Os04g49450), MYB (LOC_Os05g04210 and LOC_Os04g43680), bZIP (LOC_Os06g10880) and C2H2 (LOC_Os03g32230) which could be functionally characterized using systems biology approaches to improve our understanding of the molecular mechanism of drought tolerance.

Further, in grey module, we identified Zinc finger domain TF (LOC_Os08g06280) interacting with three genes including LOC_Os01g69070 (Auxin efflux carrier family protein), LOC_Os02g35180 (OsRR2 type-A response regulator) and LOC_Os05g06920 (Ca2+-activated RelA/spot homolog) that could be putative target genes in the drought tolerance mechanism. Zinc finger TFs have been previously reported to regulate drought tolerance in rice via regulating expression of particular target genes [34,35]. Since grey module was negatively correlated with drought stress treatment, this transcription factor may negatively regulate drought tolerance in rice. However, further characterization of this TF or its target genes using loss of function mutants would further advance our understanding of the mechanism of drought tolerance in rice [11]. Another TF identified in the present study in the turquoise module belongs to the homeobox-domain family (LOC_Os04g45810) which interacts with LOC_Os06g44190 and LOC_Os03g17790. Homeodomain transcription factors were also reported to regulate drought tolerance in rice via binding at particular cis-elements. For example, OsWox13 enhances drought tolerance in rice via regulating expression of genes involved in drought escape [31]. Thus, homeobox TF identified in our study could be a potential regulator of genes involved in drought tolerance in rice particularly those containing its cis-elements in their promoter region.

## Conclusion

We report the core drought responsive DEGs and the co-expressed modules from diverse transcriptome datasets under drought stress in rice. GO and KEGG analysis showed "sequence-specific DNA binding" and "plant hormone signal transduction" as keybiological process and metabolic pathway involved in drought tolerance in rice. Network analysis pinpointed several putative TFs from HSF, WRKY, Zinc-finger domain, homeobox domain, bZIP and MYB families that could be key regulators of drought tolerance in rice.

## Supplementary materials (available with authors):

Supplemental material 1: Primer sequences related to quantitative real-time PCR; Supplemental material 2: Samples information of different datasets; Supplemental material 3: Genes present in the different modules; Supplemental material 4: KEGG analysis to identify enriched pathways in response to drought stress; Supplemental material 5: Heatmap displaying the average log2 fold change of the expression value of selected genes from the rice core drought stress responsive genes under temporal drought stress compared to the control. Green color means down-regulation while red color means up-regulation.

## Author contributions:

Conceptualization, X.G., B.Z.; formal analysis, Y.L., L.X., K.D., K.Z., M.Z., H.X., S.T., Y.Y.; investigation, X.X.; resources, X.G., B.Z.; data curation, Y.L., L.X., K.D.; writing-original draft preparation, Y.L., L.X., K.D.; supervision, X.G., B.Z.

## Funding:

This research was funded by the National Key Research and Development Program of China, grant number 2017YFD0301500 and the Key Research and Development Projects of Hunan Science and Technology Department, grant number 2016NK2188.
